# Mindfulness program for workers in Japan: online program focused on informal training in the workplace

**DOI:** 10.3389/fpsyg.2025.1526275

**Published:** 2025-06-26

**Authors:** Mina Nakano

**Affiliations:** Department of Psychology, Fukuyama University, Fukuyama, Hiroshima, Japan

**Keywords:** mindfulness, decentering, stress-management, worker, mental health program

## Abstract

Mindfulness has gained prominence as an effective tool for managing stress and preventing depression. However, many workers in Japan find it challenging to commit to lengthy mindfulness sessions. This study addressed this issue by developing a six-session online mindfulness program tailored to Japanese workers, which emphasizes decentering (the ability to view thoughts and emotions as separate and transient) and informal mindfulness practices that can be easily incorporated into daily work routines. The program was designed to improve mental wellbeing, self-compassion, communication skills, and decentering abilities. A total of 80 participants who completed the program were compared with a control group of 134 workers who did not receive the intervention. The program comprised 90 min bi-weekly sessions held over 3 months. It incorporated brief meditation exercises and informal mindfulness practices based on actual workplace scenarios. Participants in the intervention group demonstrated significant improvements in self-compassion, communication skills, decentering, and wellbeing. Conversely, the control group showed no notable changes. These findings suggest that even short, structured mindfulness interventions can lead to substantial psychological benefits when tailored to the realities of a busy professional life.

## 1 Introduction

Workplace stress is a growing global concern, and Japan is no exception. According to the Ministry of Health Labour and Welfare (2024), 82.7% of Japanese workers report feeling anxious, worried, or stressed about their jobs. The primary sources of stress include “job failures and the burden of responsibility” (39.7%), “heavy workloads” (39.4%), and “interpersonal relationships” (including issues like power harassment and sexual harassment; 29.6%). In these high-pressure environments, many employees struggle to effectively manage their stress and maintain their mental wellbeing.

The roots of mindfulness trace back thousands of years, particularly to Buddhist traditions and other Eastern spiritual practices that emphasize present-moment awareness and non-judgmental observation of inner experiences. As Rinpoche (2007) emphasizes, the goal is not only formal meditation but also to cultivate mindfulness as a way of living. These traditional perspectives form the philosophical foundation upon which modern therapeutic and organizational applications of mindfulness are built.

Mindfulness has become prominent in recent years as an effective means of managing stress, anxiety, and depression. It has been shown to reduce the symptoms of these conditions and improve overall psychological wellbeing (Hofmann and Gómez, 2017). Mindfulness-based programs, such as mindfulness-based stress reduction (MBSR), have demonstrated positive results in various settings, including corporate environments (Janssen et al., 2018). Mindfulness has been shown to be particularly effective for stress management and overall mental health maintenance, offering individuals practical tools to cope with everyday stressors (Sharma and Rush, 2014). However, traditional programs like MBSR typically require eight sessions, each lasting nearly 3 h, as well as daily meditation practice. This can be challenging for professionals to incorporate into their busy schedules.

A key theoretical model proposed by Shapiro et al. (2006) explains how mindfulness training facilitates positive psychological changes through three interconnected mechanisms: intention, attention, and attitude. This model emphasizes that mindfulness practice requires focusing on the present moment, an intentional commitment to self-awareness, and a non-judgmental attitude toward thoughts and emotions. These mechanisms work together to shift an individual’s perspective on their inner experiences, promoting emotional regulation and stress resilience.

In addition to Shapiro et al.’s (2006) model, the foundational work by Steven C. Hayes and colleagues in developing Acceptance and Commitment Therapy (ACT) should also be acknowledged. ACT, which predates much of the modern mindfulness literature, similarly emphasizes mindfulness and acceptance as essential tools for developing psychological flexibility and reducing experiential avoidance (Hayes et al., 1999). Both frameworks highlight the importance of changing one’s relationship with thoughts and emotions rather than trying to control or eliminate them.

This shift in perspective—central to both Shapiro’s model and ACT—is closely related to the concept of decentering, a process in which individuals learn to observe their thoughts and emotions as impermanent, objective events in the mind, rather than necessarily true reflections of the self (Fresco et al., 2007). Gecht et al. (2014) suggested that mindfulness and decentering are separable concepts and that decentering can be regarded as a critical working mechanism of mindfulness. It is not sufficient to simply practice meditation; one must promote decentering. Rinpoche (2007) demonstrated that it is possible to meditate anytime, anywhere, and bringing meditation into our daily lives is one of the main objectives of Buddhist practice. Sitting for 5 or 10 min meditating a day creates an opportunity to change our perspective. By incorporating mindfulness practice into our daily lives, we can avoid being calm only during formal meditation and tense, angry, or anxious for the rest of the day, especially at work. Practicing mindfulness informally in daily life gradually eliminates the misconception that we must be somewhere peaceful to meditate and be mindful (Rinpoche, 2007). For busy workers in Japan, keeping meditation sessions short and incorporating informal practices that enhance decentering into daily life is both realistic and effective for reaping the benefits of mindfulness.

One reason mindfulness can feel disconnected from everyday life is the lack of focus on concrete communication. While existing mindfulness programs include practices like mindful listening and compassion meditation, they often fail to address real-life communication. Consequently, most of these programs do not provide practical guidance on questions such as “What is mindful behavior in this situation?”, “How can I interact with others concretely in this situation?”, and “How can I communicate without being judgmental?” It is necessary to raise awareness and establish mindful communication methods in Japanese workplaces through psychological education on mindful interpersonal communication and addressing communication challenges with mindful responses.

## 2 Methods

### 2.1 Participants

A total of 125 workers in Japan with no prior mindfulness-meditation experience from various workplaces volunteered to participate in the program. Therapists and HR personnel recruited participants by distributing a flyer through email attachments, company networks, and referrals. This study employed a non-probability sampling method, as participation was entirely voluntary. While this approach is common in workplace-based interventions due to practical constraints, its limitations regarding generalizability are acknowledged and discussed.

On average, 90 people attended each session. A questionnaire survey was conducted before and after the program, yielding 80 valid responses (16 men and 64 women, aged 22–79 years, *M* = 52.94, SD = 12.55) from participants who attended five or six sessions out of six total sessions.

The control group comprised 154 workers in Japan (55 men, 79 women, aged 23–65 years, *M* = 49.64, SD = 9.87) who only completed an online questionnaire before and after the program and who did not attend the mindfulness program. To assess group equivalence and reduce potential bias from individual differences, demographic data such as age and gender were collected and baseline comparisons were conducted across all outcome variables.

### 2.2 Program overview

This study developed and evaluated the effectiveness of a mindfulness-based mental health program designed specifically for workers in Japan. The program comprises short sessions focused on brief formal meditation practices rather than extended sessions. Additionally, it emphasizes the importance of recognizing negative thoughts and emotions that arise in the workplace and addressing them through decentering and self-compassion rather than self-blame. Through this program, mindfulness can be understood and integrated as a dedicated time for meditation as well as a practice that can be applied in everyday work life. The program is highly beneficial for busy workers, enabling them to engage in mindfulness through familiar activities connected to their daily routines.

### 2.3 Structure of the program

The program comprised six online sessions, each lasting 90 min, held weekly or bi-weekly. The program incorporated common workplace scenarios and engaged participants in mindful coping exercises to enhance decentering, communication, and self-compassion skills.

Previous interventions in the workplace, such as the 8-week, 2.5-h-per-session format used by Kersemaekers et al. (2018), demonstrated significant improvements in employee wellbeing and productivity. Compared to this, this program adopts a more time-efficient structure—90-min sessions over 3 months—balancing feasibility with theoretical and experiential learning.

The first session focused primarily on introducing the concept of mindfulness. The session explained the state in which the mind becomes consumed by thoughts and emotions in the workplace, which is contrary to the state of mindfulness. Practical examples were used to clarify concepts, deliberately avoiding overly technical language. The session also discussed the implementation of mindfulness in both international and Japanese companies, as well as its benefits from a neuroscientific perspective. The session concluded with a brief breathing meditation, and participants were assigned a daily 5-min breathing meditation exercise as homework. This session was conceptually grounded in the three-component model of mindfulness (intention, attention, and attitude) proposed by Shapiro et al. (2006).

The second session began by explaining the mindful “being mode” (a state of being here and now) and the mindless “doing mode” (a state of driven behavior; Segal et al., 2002). We emphasized that the act of consciously directing attention to routine activities in daily life using the five senses is essential for entering the “being mode.” We also discussed examples of workplace situations wherein people tend toward a state of “doing mode” and how to deal with such situations in the “being mode.” Walking meditation was introduced as an activity that can be practiced in the workplace. We introduced physical stretches that can be done in the workplace to promote awareness of one’s physical state. As homework, participants were instructed to perform a routine behavior with mindfulness in their daily life, such as mindfully eating the first bite of lunch.

The third session focuses on becoming aware of one’s thoughts and emotions, emphasizing the importance of accepting negative ones without falling into cognitive fusion—where thoughts and actual events become indistinguishable (the opposite of decentering). In an informal exercise, participants practiced observing their thoughts and emotions from a distance using the metaphor of a goldfish bowl. We also used the metaphor of cars, where participants imagined themselves standing on the street, watching thoughts and emotions, represented by passing cars, drive by without getting “inside” any of them. These exercises incorporated specific negative work situations, such as being reprimanded by a boss, feeling anxious about changing departments, or not being thanked by a colleague after offering help. For homework, participants were instructed to remember the “goldfish bowl” when a negative thought or emotion arises at work. This session is theoretically supported by the concept of decentering as outlined by Fresco et al. (2007).

The fourth session provided psychoeducation on mindful communication, including an explanation of mindful listening and its application in the workplace. We used workplace case examples to address the externalization of problems and facilitate decentering. For instance, we discussed a scenario wherein an employee who fails to meet a deadline is labeled as “sloppy.” We emphasized the importance of maintaining a non-judgmental attitude, which is essential in mindfulness. This involves listening to others without categorizing them as good or bad and fostering an attitude of acceptance by refraining from value judgments. The homework for this session was to practice mindful listening when conversing with colleagues at work. The content and approach of this session are consistent with workplace applications of mindful communication, such as those discussed by Moll et al. (2015).

The fifth session focused on the concept of decentering, exploring strategies for managing negative emotions and thoughts in the workplace, emphasizing the importance of calmly “responding” rather than reflexively “reacting.” Specific workplace scenarios were used to illustrate the difference between emotionally charged reactions and responses grounded in decentering. Participants also engaged in exercises to clarify their personal values. The session concluded with a reading of the poem “The Guest House” (Rumi, 1997), which encapsulates the essence of welcoming all emotions as part of the human experience. For homework, participants were instructed to practice responding calmly instead of reacting when negative emotions arise at work. This emphasis on values-based responding and emotional flexibility aligns with the principles of ACT (Hayes et al., 1999).

The sixth session focused on self-compassion, presenting examples of work-related situations in which individuals felt overwhelmed by feelings of self-doubt and self-blame, highlighting the absence of decentering in these instances. The session emphasized the importance of self-acceptance and healing, providing practical workplace examples on managing these emotions. We also introduced the practice of “soothing touch” placing one’s hands on one’s body—as well as brief compassion meditations, both of which can be incorporated into daily life. No homework was assigned for this final session. The theoretical framework for this session draws from Neff’s work on self-compassion (Neff, 2003).

### 2.4 Measures

#### 2.4.1 Satisfaction with the program

Participants in the intervention group were asked to rate their satisfaction with the program using a five-point scale: 1 = “Very dissatisfied”; 2 = “Somewhat dissatisfied”; 3 = “Neutral”; 4 = “Somewhat satisfied”; and 5 = “Very satisfied.”

#### 2.4.2 Self-compassion

The Japanese version of the Self-Compassionate Reactions Inventory (SCRI-J; Miyagawa and Taniguchi, 2016) was used to assess self-compassion. The scale comprises four reactions to eight hypothetical hardships. Participants select the two reactions that most closely resemble their own. A total of 16 self-compassion reactions are available for these eight items, and the self-compassion score is determined by the number of selected reactions.

#### 2.4.3 Communication skill

ENDCORE, the short version of ENDCOREs (Fujimoto and Daibo, 2007), was used to assess communication skills. It comprises six items representing important communication skills: expressivity, assertiveness, decipherer ability, other acceptance, self-control, and regulation of interpersonal relationships. The items are scored on a seven-point scale ranging from 1 = “Very bad at it” to 7 = “Very good at it.”

#### 2.4.4 Decentering

The Japanese version of the Experiences Questionnaire (J-EQ; Kurihara et al., 2011) was used to measure decentering—the state of observing thoughts and feelings as temporary events in the mind. J-EQ comprises two factors: decentering and rumination. In this study, we used decentering only, which comprises 10 items scored on a five-point scale ranging from 1 = “Never,” to 5 = “Always.”

#### 2.4.5 Mental wellbeing

The simplified Japanese version of the WHO-Five Well-Being Index (S-WHO-5-J; Inagaki et al., 2013) was used to assess mental wellbeing. It comprises five items scored on a four-point scale ranging from 1 = “Never,” to 4 = “Always.”

### 2.5 Ethical consideration

The study was approved by the Academic Research Ethics Review Committee of the author’s university (Approval No.: 2021-H-28), ensuring compliance with ethical standards in academic research. All participants provided informed consent, and the study adhered to ethical guidelines to protect participants’ privacy and confidentiality.

## 3 Results

### 3.1 Satisfaction with the program

**FIGURE 1:**
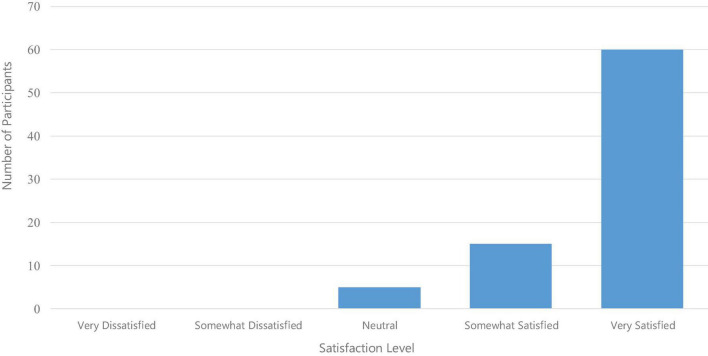
Participant satisfaction with the mindfulness program.

Participant satisfaction with the program was high (Figure 1). Of the 80 final participants, 60 (75%) were “very satisfied,” 15 (19%) were “somewhat satisfied,” and 5 (6%) were “neutral.” There were no responses indicating “somewhat dissatisfied” or “dissatisfied.”

### 3.2 Mean pre- and post-program scores for intervention and control groups

As shown in Table 1, the mean scores for the intervention group increased between the pre-test and post-test across all measures, while the control group’s scores remained relatively stable.

**TABLE 1:** Mean pre- and post-program scores and change comparisons by group.

	Group	Pre-*M* (SD)	Post-*M* (SD)	Change *M* (SD)	*t*	*p*	*d*
Self-compassion	Intervention	10.23 (4.14)	11.51 (3.37)	1.29 (3.22)	3.02	0.003	0.43
	Control	9.16 (4.53)	9.08 (4.61)	−0.08 (3.20)			
Communication skill	Intervention	24.98 (5.45)	26.53 (5.70)	1.55 (3.52)	3.46	<0.001	0.49
	Control	23.63 (6.44)	23.37 (6.28)	−0.26 (3.82)			
Decentering	Intervention	30.24 (6.31)	32.84 (5.58)	2.60 (4.47)	3.49	<0.001	0.49
	Control	29.57 (7.81)	29.43 (7.15)	−0.13 (6.10)			
Mental wellbeing	Intervention	8.16 (2.27)	9.05 (2.15)	0.89 (2.09)	2.26	0.025	0.32
	Control	6.77 (3.29)	6.90 (3.32)	0.13 (2.55)			

Change scores = post-test minus pre-test. *t*, *p*, and *d* values represent results from independent-samples *t* tests comparing intervention and control groups.

Overall, the intervention group demonstrated positive changes in self-compassion, communication skills, decentering, and mental wellbeing, compared with the control group, which exhibited minimal changes or even declining scores. While these descriptive statistics indicate the beneficial impact of the mindfulness program, the statistical significance of these changes will be examined in the subsequent analysis of variance (ANOVA; Table 2).

**TABLE 2:** Results of the two-way ANOVA with a mixed design.

	Group	Main effect of group	Main effect of time	Interaction effect
Self-compassion	Intervention	*F*(1,212) = 9.64**	*F*(1,212) = 7.06**	*F*(1,212) = 9.11**
	Control			
Communication skill	Intervention	*F*(1,212) = 7.60**	*F*(1,212) = 6.05*	*F*(1,212) = 11.95**
	Control			
Decentering	Intervention	*F*(1,212) = 5.11*	*F*(1,212) = 9.90**	*F*(1,212) = 12.17**
	Control			
Mental wellbeing	Intervention	*F*(1,212) = 21.79**	*F*(1,212) = 9.04**	*F*(1,212) = 5.08*
	Control			

*p < 0.05,

**p < 0.01.

To further support the validity of these findings, we additionally conducted independent-samples *t* tests on the change scores (post–pre) to compare the intervention and control groups directly. The intervention group showed significantly greater improvements than the control group across all variables: self-compassion [*t*(212) = 3.02, *p* = 0.003, *d* = 0.43], communication skills [*t*(212) = 3.46, *p* < 0.001, *d* = 0.49], decentering [*t*(212) = 3.49, *p* < 0.001, *d* = 0.49], and mental wellbeing [*t*(212) = 2.26, *p* = 0.025, *d* = 0.32]. These findings further confirm the efficacy of the mindfulness intervention.

### 3.3 A mixed-design two-way analysis of variance

To evaluate the effects of the mindfulness program, a mixed-design two-way ANOVA was conducted using SPSS (version 28.0). This analysis assessed the interaction between time (pre-test vs. post-test) and group (intervention vs. control group). Separate ANOVAs were performed for each of the four outcome measures: the SCRI-J, ENDCORE, J-EQ, and S-WHO-5-J. For each measure, the within-subjects factor was time (pre-test and post-test), and the between-subjects factor was group (intervention and control; Table 2).

Significant main effects were found for time and group, as well as significant interaction effects between group and time across all variables. Specifically, self-compassion, communication, decentering skills, and mental wellbeing significantly increased in the intervention group from pre- to post-program, whereas no significant changes were observed in the control group. These results indicate that the intervention group demonstrated significant improvements over time compared with the control group.

## 4 Discussion

In this study, we developed a mindfulness-based mental health program, tailored specifically for workers in Japan, and evaluated its effectiveness through various psychological measures. The program was designed with busy workers in mind, offering short, manageable sessions focusing on brief formal meditation practices. In addition to these meditative exercises, the program emphasized recognizing and addressing the negative thoughts and emotions that arise in the workplace using decentering and self-compassion techniques. This approach encouraged participants to respond mindfully to challenges rather than reacting with self-blame or judgment toward themselves or others. The program’s goal was to demonstrate how mindfulness can be applied during dedicated meditation times as well as throughout daily work life, particularly in moments of stress or interpersonal conflict. By integrating mindfulness into everyday activities, the program aimed to provide practical tools that could be easily incorporated into the participants’ routines, promoting mental wellbeing and greater resilience in the workplace.

Although the duration of formal exercises in each program session, such as breathing meditation, was under 10 min, this brevity did not appear to negatively impact participant satisfaction; 94% of participants reported being “very satisfied” or “somewhat satisfied” with the program. This finding is significant, as it suggests that the success of mindfulness interventions may not depend solely on the length of formal meditation practices. Instead, integrating informal mindfulness practices—such as mindful awareness during daily activities—and structured educational components on mindful coping skills for managing thoughts, emotions, and communication, may be critical for fostering overall wellbeing. Similar findings have been reported in previous studies demonstrating that brief, workplace-integrated mindfulness programs can be effective in reducing stress and enhancing wellbeing (Arredondo et al., 2017; Healy et al., 2024).

The results of this study highlight the potential benefits of incorporating informal mindfulness practices, such as mindful communication or responding mindfully to workplace challenges, into daily life. Such practices enable participants to apply mindfulness principles beyond structured meditation sessions. This real-world application may help participants more effectively decenter from stressful thoughts and emotions, enhancing their capacity to manage work-related stressors. These findings are consistent with previous research showing that increases in mindfulness and self-compassion mediate reductions in stress and fatigue among working adults (Van der Meulen et al., 2020).

Moreover, while formal meditation is essential to mindfulness, this study suggests that it is not the sole driver of the observed positive outcomes. By providing psychoeducation on mindful listening, decentering, and self-compassion, participants gained both meditation tools and practical skills for interpersonal communication and emotional regulation. This holistic approach may be especially beneficial in workplace settings, wherein interactions with colleagues and emotional management are critical to performance and satisfaction.

The improvement in self-compassion scores also suggests that short, accessible mindfulness practices can significantly affect how individuals relate to themselves. This is crucial in the context of workplace stress, wherein self-criticism and rumination often exacerbate stress. Our findings are consistent with previous studies demonstrating that brief mindfulness-based interventions can effectively reduce self-criticism and enhance self-compassion (Halamová et al., 2018; Lancheros et al., 2023; Ondrejková et al., 2020). These results support the idea that fostering self-compassion through brief, structured interventions may be a key mechanism for mitigating workplace-related psychological distress.

Compared to previous studies, this program presents several distinctive features. Arredondo et al. (2017), for example, focused on healthcare professionals and implemented a more intensive, clinically oriented mindfulness and self-compassion training. While their findings were promising, the format may not be easily transferable to broader workplace settings. Healy et al. (2024) developed a brief compassion-based program for school employees; although effective, their intervention was limited in scope and target population. In contrast, the present study offers a psychoeducational, multi-component program—emphasizing informal practices, decentering, and mindful communication—adapted for diverse occupational settings and time constraints. These distinctions highlight the practical and scalable nature of this intervention for real-world workplaces.

A relevant comparison can also be made with longer-format mindfulness interventions in workplace settings. For example, Kersemaekers et al. (2018) implemented a mindfulness program for employees that involved 2.5-h sessions conducted weekly over an 8-week period. Their study found significant improvements in wellbeing, emotional regulation, and interpersonal functioning. While such programs are effective, the intensive time commitment may pose practical challenges for widespread adoption in typical organizational contexts. In contrast, the present study demonstrates that similar psychological benefits—such as improvements in self-compassion, communication skills, and decentering—can be achieved through a shorter, more flexible format. This finding suggests that brief, structured mindfulness interventions may serve as a viable alternative when longer sessions are not feasible.

A key implication of this study is the role of mindfulness in fostering long-term behavioral change. By integrating mindfulness principles—such as mindful listening or being present during routine tasks—into everyday activities, participants could likely sustain the program’s benefits over time. This indicates the importance of viewing mindfulness as a set of meditative exercises and an approach to life. Previous studies have highlighted that sustained improvements in emotional regulation and psychological resilience are more likely when mindfulness is integrated into daily life through informal practices (Fresco et al., 2007; Van der Meulen et al., 2020). Embedding mindfulness into daily practices helps create lasting changes in attitudes and behaviors, thereby promoting resilience and improved wellbeing.

A key strength of this study lies in its practical applicability. The mindfulness program was specifically designed to fit within the time constraints of busy employees by offering short, online sessions that emphasize informal practices integrated into daily work routines. This format enhances both accessibility and feasibility, making it a realistic and scalable approach to improving employee wellbeing in actual workplace settings. By reducing barriers to participation, such as time commitment and the need for formal meditation experience, the program demonstrates how mindfulness can be meaningfully adapted to organizational environments.

Beyond individual-level outcomes, this study has important managerial and social implications. From a managerial standpoint, the results suggest that brief, accessible mindfulness programs can be incorporated into workplace wellness strategies without significantly disrupting operations. By fostering greater self-awareness, emotional regulation, and mindful communication, such interventions may support leadership development, enhance teamwork, and contribute to healthier organizational climates. From a broader social perspective, the success of this flexible and time-efficient approach highlights the feasibility of scaling up mindfulness-based mental health promotion in diverse occupational settings. This has relevance for policymakers and public health initiatives aiming to reduce workplace stress and promote psychological resilience across the working population.

## 5 Limitations

While this study demonstrates significant short-term improvements in self-compassion, communication skills, decentering, and mental wellbeing, several limitations should be acknowledged.

### 5.1 Participant-related limitations

The sample may be subject to recruitment bias, as participation was voluntary. This could reflect a greater intrinsic interest in mindfulness or mental health among the intervention group. Additionally, the sample was predominantly female and modest in size (*n* = 80 for the intervention group), which limits the generalizability of findings and the ability to detect subgroup differences. Although demographic data such as age and gender were collected, the sample sizes within subgroups were insufficient to conduct reliable between-group analyses. Future studies should aim for more diverse and demographically balanced samples to examine potential moderators such as age, gender, or occupational role.

### 5.2 Methodological limitations

Outcomes were assessed only immediately after the intervention and relied solely on self-report questionnaires. While these measures captured participant experiences, they are susceptible to biases like social desirability or cognitive dissonance. The absence of follow-up assessments also limits conclusions about the sustainability of observed effects. Incorporating longitudinal designs and objective indicators—such as physiological markers or third-party ratings—would provide a more robust understanding of program impact.

### 5.3 Design and scope limitations

A significant baseline difference in mental wellbeing scores was identified between groups, with the intervention group starting at a higher level. Although change scores and mixed ANOVA were used to mitigate this issue, the imbalance may still confound results. Moreover, while improvements were observed at the individual level, this study did not examine outcomes at the organizational level, such as team functioning or job satisfaction. Evaluating these broader impacts would help determine whether such interventions yield benefits beyond the individual.

In summary, these limitations underscore the need for future research employing larger and more representative samples, longitudinal designs, objective outcome measures, and assessments of workplace-level effects to build upon the findings of this study.

## Data Availability

The raw data supporting the conclusions of this article will be made available by the authors, without undue reservation.
